# Are Patients Traveling for Intraoperative Radiation Therapy?

**DOI:** 10.1155/2017/6395712

**Published:** 2017-10-09

**Authors:** Kelsey E. Larson, Stephanie A. Valente, Chirag Shah, Rahul D. Tendulkar, Sheen Cherian, Courtney Yanda, Chao Tu, Jessica Echle, Stephen R. Grobmyer

**Affiliations:** ^1^Division of Breast Services, Department of Surgery, Cleveland Clinic, Cleveland, OH, USA; ^2^Division of Breast Surgery, Department of Surgery, University of Kansas, Kansas City, KS, USA; ^3^Department of Radiation Oncology, Cleveland Clinic, Cleveland, OH, USA; ^4^Quantitative Health Science, Cleveland Clinic, Cleveland, OH, USA

## Abstract

**Purpose:**

One benefit of intraoperative radiation therapy (IORT) is that it usually requires a single treatment, thus potentially eliminating distance as a barrier to receipt of whole breast irradiation. The aim of this study was to evaluate the distance traveled by IORT patients at our institution.

**Methods:**

Our institutional prospective registry was used to identify IORT patients from 10/2011 to 2/2017. Patient's home zip code was compared to institution zip code to determine travel distance. Characteristics of local (<50 miles), regional (50–100 miles), and faraway (>100 miles) patients were compared.

**Results:**

150 were patients included with a median travel distance of 27 miles and mean travel distance of 121 miles. Most were local (68.7%), with the second largest group living faraway (20.0%). Subset analysis of local patients demonstrated 20.4% traveled <10 miles, 34.0% traveled 10–20 miles, and 45.6% traveled 20–50 miles. Six patients traveled >1000 miles. The local, regional, and faraway patients did not differ with respect to age, race, tumor characteristics, or whole breast irradiation.

**Conclusions:**

Breast cancer patients are traveling for IORT, with 63% traveling >20 miles for care. IORT is an excellent strategy to promote breast conservation in selected patients, particularly those who live remote from a radiation facility.

## 1. Introduction

Over the last 10 years, an increasing number of breast cancer patients are receiving intraoperative radiation therapy (IORT) [1]. One of the major benefits of IORT is the delivery of radiation in a single setting rather than multiple visits over several weeks as required for whole breast irradiation (WBI). This time consideration is very important for patients as traveling for daily WBI has been shown to have a significant impact on psychological, financial, work, and social aspects of their lives [2, 3].

In addition, prior studies have demonstrated that rates of breast conservation and radiation therapy compliance are inversely related to patients' proximity to a radiation facility. These associations have been shown at state and national levels, as well as on an international scale [4–9]. Travel distance continues to be a barrier to care which is not improving with time, despite increasing numbers of hospitals and radiation centers across the United States [10]. The shortened radiation course offered by IORT has the potential to decrease this barrier to care and promote breast conservation in individuals who live far from a radiation facility and cannot travel for daily radiation treatments.

To the author's knowledge, the distance traveled by patients who undergo IORT in the United States has not previously been assessed. The aim of this study was to evaluate the average travel distance for patients treated with IORT at our institution, to assess patient and tumor factors associated with increased travel distance, and to determine if the distance traveled by patients changed over time.

## 2. Materials and Methods

Patients who undergo IORT at our institution are enrolled in an IRB-approved prospective data registry. Patient information is entered and maintained by the surgeon, radiation oncologist, and research coordinators. Candidates for IORT are identified after multidisciplinary consultation and treated with the Zeiss Intrabeam system at the time of their initial breast cancer operation. Preplanned IORT boost is not routinely offered. The addition of WBI following surgery is determined after multidisciplinary discussion upon review of the final surgical pathology.

The institution registry was used to identify patients who underwent IORT from 10/2011 to 2/2017. Data obtained included patient demographics, tumor characteristics, and adjuvant therapy (chemotherapy and WBI). Patient income, education, employment, marital status, and insurance status have been associated with receipt of radiation in prior studies; however, these factors are not included in the registry and thus were not available for analysis. To determine travel distance, the patient's home zip code was compared to the institutional zip code using Google Maps. The shortest route was recorded as the patient's travel distance, in miles.

When designing the study, we purposefully elected to not include a comparison group of WBI patients. Our institution has numerous excellent WBI treatment centers throughout the region such that when WBI patients are seen at our tertiary facility for initial consultation, their actual WBI care is often coordinated purposefully at the closest WBI location to their home or work. Thus the travel distance for WBI patients at our institution is potentially confounded by bias with this purposeful redistribution of treatment locations, which could skew the results of a side-by-side comparison of IORT and WBI patients. With this in mind, we felt it was most sound to instead provide a detailed analysis of the IORT patients' travel distance and avoid potential for bias that would be introduced with a WBI comparison group.

For analysis, patients were subdivided into three groups: local (<50 miles), regional (50–100 miles), and faraway (>100 miles). The groups were compared to determine any differences. Patient characteristics in each group were reported as counts and percentages or mean with standard deviation, where appropriate. After comparing patient groups by travel distance, the median and average distance traveled by treatment year were compared to determine any trends over time. For analysis, two-tailed Chi-squared and Fisher's exact tests [R software (v3.31, 2016-0-21)] were used with *p* value < 0.05 being considered statistically significant.

## 3. Results

Registry review identified 150 women for study inclusion. The average age was 70.8 years, and most were Caucasian (89.3%). The average tumor size was 1.0 cm, with most patients having ER positive (99.3%), PR positive (87.3%), HER2 negative (99.3%), invasive ductal cancers (60.6%), and N0 disease (99.3%). Only 3% of patients received adjuvant chemotherapy. Three individuals (2%) had adjuvant WBI due to a close margin (*n* = 1), positive sentinel node on final pathology (*n* = 1), and multifocal disease in the surgical specimen (*n* = 1).

The median travel distance for all patients from home to the treatment facility was 27 miles. The mean travel distance was 121 ± 284 miles. Most patients were local (*n* = 103, 68.7%), with the second largest group living >100 miles away (*n* = 30, 20.0%). The remaining 17 patients (11.3%) traveled from a regional distance for treatment. Subset analysis of the local group indicates that 21 patients (20.4%) traveled <10 miles, 35 patients (34.0%) traveled 10–20 miles, and 47 patients (45.6%) traveled 20–50 miles. Thus, the majority of IORT patients (94/150, 63%) traveled at least 20 miles for treatment. In the faraway group, six patients (4%) traveled more than 1000 miles.

The three distance groups did not differ with respect to age, race, tumor characteristics, or adjuvant WBI (Table 1). However, the groups did differ with respect to adjuvant chemotherapy with patients living faraway receiving adjuvant chemotherapy more frequently despite similar tumor profiles between the groups (Table 1).

When comparing the travel distances by treatment year, there was no difference in median or mean distance traveled for patients treated across the years (Table 2).

## 4. Discussion

Breast cancer patients who undergo IORT are commonly traveling for treatment, with 63% of patients in this series traveling more than 20 miles for care. The national average distance that breast cancer patients travel to the nearest radiation facility is 4.8 miles [4, 11], and drop-off in breast conservation rates and radiation treatment use have been documented once patients must travel more than 9–25 miles [4, 10]. The median distance traveled by IORT patients in this study (27 miles) is more than 5x the previously reported average distance for individuals receiving WBI, indicating patients are willing to travel for the benefits offered by IORT.

The majority of patients in this study passed multiple other breast cancer centers along the way to our institution, including six National Accreditation Program for Breast Centers (NAPBC) sites within a 20-mile radius [12]. This documented increased travel distance is in striking contrast to a recent publication which showed that patients frequently choose lower-volume institutions for care in order to shorten travel distance, despite worse outcomes [13]. As our institution is the only center to offer IORT within this radius, we suspect the availability of this technology may in part attribute to the large travel distances documented in this study. There were no patient or tumor factors identified in our study which were linked to travel distance so further research would be required to delineate socioeconomic or psychologic factors which may impact patient decision regarding travel for IORT.

Patients presenting to our institution commonly inquire about IORT and its potential benefits, including eliminating recurrent visits for radiation treatment. Coombs et al. documented significantly reduced travel time as well as significantly improved environmental impact for patients who undergo IORT in the UK [2]. In their study, the average travel time for WBI was 14 hours versus 3 hours for IORT patients. In TARGIT-R, the largest study to evaluate IORT use in North America, most patients (65.4%) underwent IORT as single-dose treatment at the time of their breast cancer operation [1], potentially saving 4–6 weeks of travel for WBI per patient. Relatively fewer individuals received IORT as a boost (27.4%) [1], potentially saving these patients one week of travel for WBI boost. In our study, an even higher rate of patients (147/150, 98.0%) required only single-dose IORT during their operation, thus reflecting an even greater relative time and travel distance savings per patient, with the lower rates of WBI likely due to careful patient selection.

Across time, the geographic distribution of IORT patients remained stable as reflected in the median and mean travel distance (Table 2). For this analysis, only 2014–2016 data was included to represent established years for the IORT program at our institution. The remaining years of patients were excluded due to low patient numbers as the IORT program was starting (2011–2013) and only partial year of data available based on the timing of analysis (2017). Going forward, the authors suspect that distance traveled by IORT patients will increase as more patients and providers learn about this technology and seek it out for individualized breast cancer care.

Complete financial analysis from patient and institution levels was outside the scope of this manuscript but would be a potential area of interest in future studies. Financial considerations for the patient may include cost of travel, cost of missing work or school for multiple radiation visits, or potential copayment/insurance costs for single versus multiple visit treatment protocols. Future studies may also help to clarify what role, if any, financial considerations played for patients who had increased travel distance as documented in this study.

In summary, our study is the first to assess travel distance for IORT patients in the United States and demonstrates that patients are traveling for the benefits offered by IORT. It is important for clinicians to recognize the potential for IORT to decrease travel burden, promote breast conservation and radiation compliance, and eliminate a major barrier to care for appropriately selected breast cancer patients.

## 5. Conclusion

Breast cancer patients are traveling for the benefits offered by IORT, with 63% of patients traveling more than 20 miles for treatment. IORT is an excellent strategy to help decrease a barrier to care (distance), thus promoting breast conservation in selected patients who live remote to a radiation facility or those who cannot commit to daily radiation treatments.

## Figures and Tables

**Table 1:** Comparison of patients who live local (<50 miles), regional (50–100 miles), and faraway from the treating institution.

	Local patients (<50 miles) *n* = 103 (68.7%)	Regional patients (50–100 miles) *n* = 17 (11.3%)	Faraway patients (>100 miles) *n* = 30 (20.0%)	*p* value
Mean travel distance (miles)	19.8	70.0	500.6	<0.001^*∗*^

Age (years)	71.3	71.8	68.6	0.28

Race				
Caucasian	90 (90%)	17 (100%)	27 (93.1%)	0.34
African American	8 (8%)	0	0 (0%)	
Asian	1 (1%)	0	1 (3.5%)	
Other	1 (1%)	0	1 (3.5%)	

Tumor type				
IDC	63 (61.2%)	11 (64.7%)	17 (56.7%)	0.41
ILC	4 (3.8%)	0	1 (3.3%)	
Mixed	35 (34.0%)	5 (29.4%)	10 (33.3%)	
Other	1 (1%)	1 (5.9%)	2 (6.6%)	

ER positive	103 (100%)	17 (100%)	29 (96.7%)	0.31

PR positive	88 (85.4%)	15 (88.2%)	27 (93.1%)	0.59

HER2 positive	1 (1%)	0	0	0.34

Adjuvant chemotherapy	0	0	4 (14.3%)	0.002^*∗*^

Whole breast radiation	2 (2%)	0	1 (3.6%)	0.67

^*∗*^<0.05 = significant.

**Table 2:** Travel distances (in miles) by year.

	2014 *N* = 31	2015 *N* = 42	2016 *N* = 45	*p* value
Median travel distance	23	33	27	0.17
Mean ± standard deviation travel distance	99 ± 281	102 ± 216	99 ± 220	0.99
